# Probing the druggability of membrane-bound Rab5 by molecular dynamics simulations

**DOI:** 10.1080/14756366.2016.1260564

**Published:** 2017-01-16

**Authors:** Eileen Edler, Matthias Stein

**Affiliations:** Molecular Simulations and Design Group, Max Planck Institute for Dynamics of Complex Technical Systems, Magdeburg, Germany

**Keywords:** Small GTPase, protein-membrane association, post-translational modification, geranylgeranylation, lipid anchoring

## Abstract

Rab5 is a small GTPase and a key regulator in early endosomal trafficking. Rab5 and its effectors are involved in a large number of infectious diseases and certain types of cancer. We performed µs atomistic molecular dynamics simulations of inactive and active full-length Rab5 anchored to a complex model bilayer with composition of the early endosome membrane. Direct interactions between the Rab5 G domain and the bilayer were observed. We found two dominant nucleotide-dependent orientations characterised by a different accessibility of the switch regions. The “buried switch” orientation was mainly associated with inactive Rab5 accompanied with a rather extended structure of the hypervariable C-terminal region. Active Rab5 preferred an orientation in which the switch regions are accessible to effector proteins. These structural differences may provide an opportunity to selectively target one Rab5 state and lead to new approaches in the development of Rab5-specific therapies.

## Introduction

The Rab family is one of the five subfamilies of the Ras superfamily of small GTPases^1^ and is representing the most abundant family in humans^2^ with more than 60 members. Rab proteins can be classified into eight^3^ or nine^4^ different functional groups. Recently, Stein et al. classified 62 human Rab proteins according to their distances in electrostatic potentials and identified six subclusters with conserved electrostatic potentials^5^. Rab proteins are key regulators of membrane organisation and trafficking and mediate membrane signalling through their selective and unambiguous assignment to specific membrane compartments^6^^,^^7^. Like other members of the Ras superfamily, Rab proteins are molecular switches being present in either an inactive (GDP-bound) or an active (GTP-bound) state, in the following Rab(GDP) or Rab(GTP), respectively.

**Figure 1.**
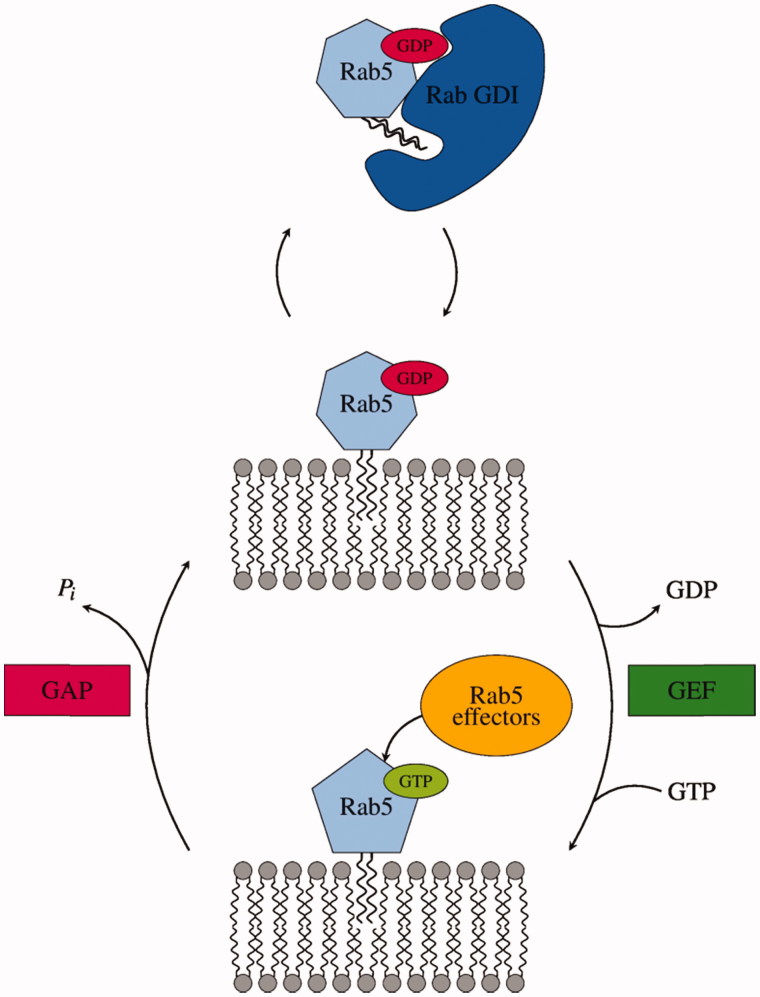
Catalytic cycle and intracellular localisation of Rab5. Small GTPases switch into an active state through the exchange from GDP to GTP mediated by guanine nucleotide exchange factors (GEFs). GTPase-activating proteins (GAPs) catalyse the hydrolysis of GTP to GDP, thereby restoring the GTPase inactive state. Effector proteins exclusively bind Rab5(GTP).

Rab(GDP) shuttles between the cytoplasm and the membrane surface, whereas Rab(GTP) localisation is restricted to the bilayer surface. A striking feature of the Rab GTPase family is the distinct intracellular localisation patterns of different Rab members which is still not completely understood. Thus, Rab GTPases enable a temporal as well as spatial control of transport processes^6^. Membrane anchoring is realised via two post-translationally attached geranylgeranyl lipid moieties at the protein C-terminus (Figure 1). Rab(GTP) recruits a variety of specific regulatory and effector proteins to the membrane surface, thereby mediating processes in cargo transport like vesicle budding, tethering and fusion^4^.

Interactions of Rab GTPases with various effectors as well as crosstalk among Rab proteins make them a central focal point of cellular trafficking, recycling and degradation processes. The physiological relevance of Rab GTPases is based on their central role in membrane trafficking. Thus, it is not surprising that Rab GTPases and their regulators or effectors are associated with many diseases. In particular, infectious, neurological and endocrinological diseases may result from pathogen-induced or inherited dysfunctions of Rab pathways. Controlling parts of the innate immune response via phagosome maturation, Rab protein-dependent mechanisms are tackled by viruses and intracellular bacteria to prevent their degradation in phagolysosomes. The involvement of Rab proteins in cell signalling leads to an assignment of their dysfunctions with different types of cancers^4^.

In this work we focus on one representative of Rab proteins, the early endosome marker protein Rab5. It is a small GTPase associated with early endosomal trafficking, regulating clathrin-mediated endocytosis. In the immune system Rab5 regulates phagocytic transport and the maturation of phagosomes, a process that eventually leads to fusion with lysosomes and degradation of pathogens. Bacteria like *Listeria monocytogenes* try to modify the function of Rab5 and other Rab GTPases in order to evade the degradation pathway and escape from phagosomes to subsequently grow uncontrollably in the cytoplasm. *L. monocytogenes* has been reported to impede the Rab5(GDP) to Rab5(GTP) exchange with the result that the fusion with lysosomes is prevented. However, the exact mechanism by which Rab5 nucleotide exchange is inhibited is still unknown^8^. Rab5 is also responsible for controlling events in macropinocytosis, i.e. the process of solute and fluid uptake. The flow of substances through polarised epithelial cells is regulated by protein complexes called tight junctions. The group B coxsackie virus is known to internalise tight junctions to escape into the cytoplasm in a Rab5- and Rab34-dependent manner. Knockout experiments with siRNA as well as mutational studies have shown that functional Rab5 is required for virus internalisation; however, constitutively active and inactive Rab5 block virus infection^9^.

In the early endosome membrane, Rab5 and its effector proteins establish a phospholipid signalling platform that contains significant amounts of PI(3)P (phosphatidylinositol 3-phosphate) in a positive feedback loop^6^. Similarly, Rab5 was shown to be involved in the formation of PI(3)P on *Salmonella*-containing vacuoles (SCVs) during infection with *Salmonella enterica serovar typhimurium*^10^. Fusion of Rab5-positive vesicles with SCVs is dependent on the phosphatase activity of bacterial SopB protein, leading to the recruitment of the Rab5 effector Vps34, subsequent formation of PI(3)P and SCV maturation. The exact mechanism on how dephosphorylation of PI(4,5)P_2_ (phosphatidylinositol 4,5-bisphosphate) promotes Rab5 recruitment remains unclear. Mallo et al. speculate that electrostatic interactions, known to influence Rab GTPase localisation, may play a role^10^. Depletion of PI(4,5)P_2_ on the so-called prevacuoles is also described to be a prerequisite in *Yersinia pseudotuberculosis* entry^11^. The bacterium resides in PI(4,5)P_2_-rich prevacuoles which can only be released into the host cell when PI(4,5)P_2_ is hydrolysed. Dephosphorylation of PI(4,5)P_2_ and therefore membrane scission of the prevacuole is realised via the recruitment of Rab5-positive vesicles and the Rab5 effector phosphatases OCRL (oculocerebrorenal syndrome of Lowe protein) and INPP5B (inositol polyphosphate-5-phosphatase B). Besides, its importance in clathrin-mediated endocytosis, phagocytosis and macropinocytosis, Rab5 was also found to be associated with the formation of invadosomes in human breast cancer^12^. Overexpression of Rab5 triggers tumour invasion, metastasis, and dissemination to further organs in a Rab4-dependent manner.

Rab proteins are closely related to the Ras small GTPases, which represent the classic example for oncogenes involved in various types of human cancer. Although the effects of single mutations in Rab proteins or effectors could not be characterised in Alzheimer disease^13^, an upregulation of Rab5 and Rab7 has been observed. Recent work has identified downstream regulation of Rab5 to be associated with Huntington’s disease^14^, and Parkinson (for a review, see work by Mitra et al.^15^). Thus, an investigation of the molecular details of endosomal trafficking provide biophysical insight, help to identify possible drug targets and then lead to an effective treatment of the above-mentioned diseases.

Approaches to modify Rab5 signalling may follow the therapeutic approaches to inhibit Ras signalling, for a review see^16^. Understanding the molecular mechanisms underlying Rab5 function in healthy cells, however, allows first insight on how pathogens are able to regulate Rab5 and its downstream effectors for their own purposes. MD simulations have been shown to be a valuable tool for the examination of membrane-associated proteins and small peptides^17^^,^^18^. Comprehensive MD studies have been performed on K-Ras^19^ and N-Ras C-terminal peptides^20–22^ bound to model membranes and were compared with experimental NMR spectroscopy data. Furthermore, MD simulations were performed on lipidated full-length H-Ras in an uncharged membrane^23^ and K-Ras protein bound to an anionic bilayer^24^. They found that membrane association is not only mediated by the protein lipid anchors but also partly by interaction of amino acid side chains in the catalytic domain with the lipid phosphates (see current findings reviewed in^25^).

The term “druggability” of a target protein describes the accessibility of a small-molecule drug to a binding site and is a necessity for a successful transformation from a ‘hit’ to a ‘lead’ compound^26^.

We here present first full-atomistic Molecular Dynamics (MD) simulations to investigate the membrane localisation and orientation of post-translationally modified, full-length Rab5(GDP) and Rab5(GTP) proteins. MD simulations revealed an interaction of the protein G domain with the membrane surface in both activation states. This was accompanied by a rotation around the protein axis and a re-orientation of the switch regions, which can then be recognised by regulatory and effector proteins. In Rab5(GDP) the switch regions adopt a “syn” conformation being partially buried between the protein and the membrane surface and are therefore not accessible for binding partners. The long hypervariable C-terminal region, that represents a linker between the protein core and the membrane–anchor geranylgeranyl chains, adopts an extended structure favourable for protein–protein interactions. In contrast, the switch regions of Rab5(GTP) remain in an almost “anti” conformation solvent- and binding partner accessible. This makes only Rab(GTP) state accessible to potential small molecule inhibitors.

## Material and methods

### Preparation of Rab5 protein and membrane structural models

Rab5 consists of 215 amino acid residues. Rab5 association with the membrane is realised via two-fold lipidation of adjacent cysteine residues at the protein C-terminal end, which are modified by one geranylgeranyl (GG) unsaturated hydrocarbon chain each. The hypervariable region is approximately 30 amino acids long and connects this lipid anchor to the catalytic G domain (residues 15–184). This linker region as well as a 15 amino acids N-terminal region are highly dynamic and could not be resolved in X-diffraction experiments. Available crystal structures comprise only the G domain and lack the hypervariable region required for membrane binding. We used the PDB entries 1TU4^27^ and 1R2Q^28^ as starting structures for Rab5(GDP) and Rab5(GTP) models, respectively. Originally phosphoaminophosphonic acid-guanylate ester (GNP), i.e. an analogue of GTP, was bound to Rab5 in 1R2Q, which was replaced by GTP in our simulations. We predicted models of full-length Rab5 structures using the MODELLER 9.12 software^29^. The following preparatory steps were performed individually for both GDP- and GTP-bound Rab5 states. Ten different structural models for the N- and C-terminal regions were created keeping the structure of the G domain fixed. The quality of the models was estimated with the Discrete Optimised Protein Energy (DOPE) score^30^ and the model with the lowest DOPE score was chosen for further refinement. A subsequent torsional sampling conformational search was performed with MacroModel v10.0^31^ and the OPLS-2005 force field^32^. Structures obtained from this sampling procedure were clustered based on their root mean square deviations (RMSDs), and the lowest energy model, which was closest to the average structure of the largest cluster, was chosen. The full-length Rab5 structure was then solvated with explicit water and modelled for 50 ns with the G domain kept fixed. Next, the GG chains were attached to the Cys212 and Cys213 residues at the protein C-terminus. The final model was again solvated, energy minimised and modelled at 310 K in a fully unconstrained 50 ns Molecular Dynamics (MD) simulation.

The model membrane was generated using the CHARMM-GUI Membrane Builder^33^, followed by subsequent energy minimisation, heating to a temperature of 310 K and a 50 ns MD simulation in order to equilibrate the bilayer. The lipid composition was chosen in order to emulate the early endosome membrane which resembles the plasma membrane but features higher amounts of PI(3)P. The lipid types and exact numbers of lipids are shown in Table 1 and are equal for both leaflets (symmetric membrane). Membrane structural parameters such as lipid area and membrane thickness were carefully monitored in order to ensure membrane equilibration (see Figure S1, Supplementary Information).

**Table 1.** Composition of the model membrane used in the MD simulations. POPS and PI(3)P carry negative charges and render the whole membrane charge negative.

Lipid type	Number of lipidsin per leaflet	Ratio
Palmitoyl-oleoyl-phosphatidylcholine (POPC)	90	17.8%
Cholesterol (CHOL)	150	29.7%
Palmitoyl-sphingomyelin (PSM)	50	9.9%
Palmitoyl-oleoyl-phosphatidylethanolamine (POPE)	135	26.7%
Palmitoyl-oleoyl-phosphatidylserine (POPS)	55	10.9%
Phosphatidylinositol 3-phosphate (PI(3)P)	25	5.0%

The lateral dimensions of the bilayer were 16.9 nm × 16.6 nm. The protein GG anchor was manually inserted into the membrane layer by replacing one POPC lipid molecule.

### Molecular dynamics simulations – computational details

All MD simulations were performed with NAMD2.9^34^ and the full-atomistic CHARMM36 force fields for proteins and lipids^35–38^. The parameters for the nucleotides GDP and GTP were adapted from the established parameter set for nucleic acids^39^ and combined with phosphate group parameters from ADP/ATP^40^. The topology of the geranylgeranyl (GG) post-translationally modified cysteine residues was derived from a combination of existing protein and lipid topologies.

Solvent interactions were modelled using the explicit TIP3P water model^41^. In preparation of the MD production runs, the membrane–protein–water system was energy minimised, heated to 310 K and equilibrated for 50 ns. For both Rab5(GDP) and Rab5(GTP), three independent MD simulations were performed with a total production simulation time of 1.35 µs for each nucleotide state (3 × 450 ns). The production runs were performed in an ensemble of constant number of particles, pressure and temperature, controlled by Langevin dynamics^42^. Periodic boundary conditions were applied and the Particle Mesh Ewald (PME)^43^ method was used to calculate electrostatic interactions. Positions, velocities, and forces were calculated every 2 fs. The Cα atom root mean square fluctuation (RMSF) was computed after superposing the G domain coordinates onto the first frame structure by using the VMD “measure fit” and “measure rmsf” commands.

## Results

**Figure 2.**
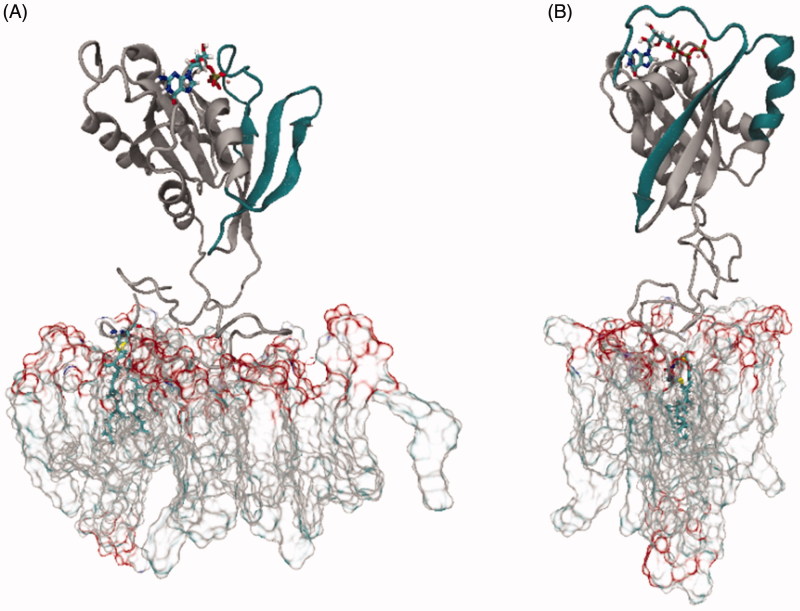
Starting configurations of membrane-bound Rab5(GDP) (A) and Rab5(GTP) (B). The switch I and switch II regions are coloured in dark petrol.

All MD simulations, whether GDP- or GTP-bound, started from a perpendicular position of Rab5 relative to the membrane surface (Figure 2).

**Figure 3.**
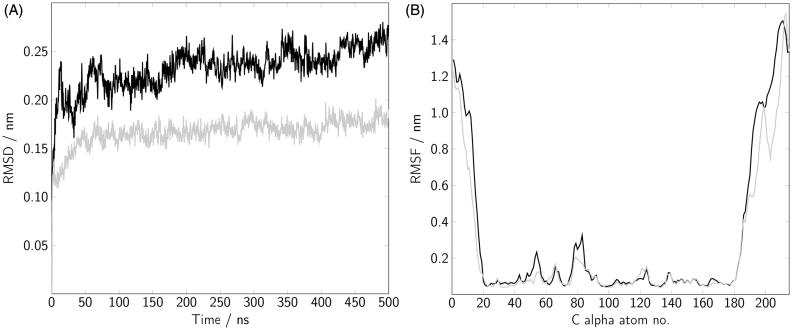
(A) The root mean square deviation (RMSD) of the Rab5 G domain Cα atoms is shown for a 500 ns MD trajectory including 50 ns of equilibration. (B) The root mean square fluctuations (RMSF) were calculated after superposing the G domain coordinates in each frame to the first frame structure. Data for Rab(GDP) (black) and Rab(GTP) (gray) is averaged over three independent runs.

The root mean square deviation (RMSD) of the Cα atoms of the G domain was calculated for the complete 500 ns trajectory for each MD simulation including a short equilibration period (Figure 3(A)). Data from the three independent runs of Rab(GDP) and Rab(GTP) were averaged to allow activation state-dependent statements. After ∼50 ns the RMSD started to fluctuate around a value of 0.24 and 0.17 nm for GDP- and GTP-bound Rab5, respectively, and afterwards increased only slightly. In order to identify protein regions of low and high stability the RMSF was calculated over the last 450 ns (Figure 3(B)). Comparing Rab5(GDP) and Rab5(GTP), the RMSF profiles of the G domains exhibit largest structural deviations around the switch I (residues 44–66) and the switch II (residues 75–91) regions. These regions are essential for binding effector proteins and the nucleotide in both Rab5 states. However, fluctuations in the Rab(GDP) switch regions are slightly more pronounced. As expected, the N- and C-terminal regions are significantly more flexible than the G domain. The decrease of flexibility at the C-terminus (residues 208–215) is a consequence of interactions with membrane lipids which stabilise the position of these residues (see below).

**Figure 4.**
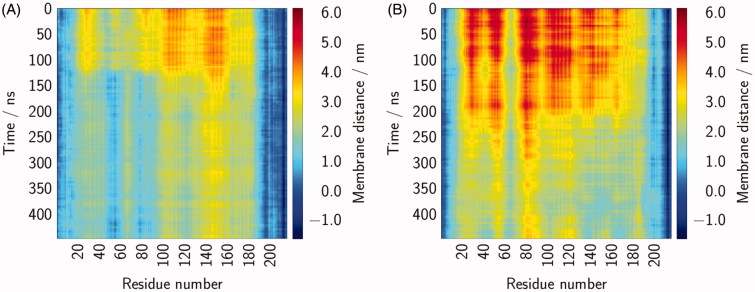
The protein–membrane distances were calculated for the different Rab5 amino acids over 450 ns. Data were averaged over three runs for both Rab(GDP) (A) and Rab(GTP) (B).

In order to characterise the dynamics of Rab5 more in detail, different structural parameters were defined, whose temporal development was investigated. The distance between the bilayer and the protein was measured as the z-distance between the phospholipid P atoms and the centre of mass of the single amino acids. The absolute protein–membrane distance was at a maximum in the beginning and decreased over the simulated time of 450 ns as the protein structures tilted towards the bilayer. For Rab(GDP) the residues 105–120 and 142–157 were at largest distance from the bilayer surface with values of 4.5 nm above the membrane surface (Figure 4(A)). During the simulations the whole protein approached the bilayer; a clear bending was observed after ∼120 ns. Residues 142–157 form an α-helix which kept the largest distance with ∼2.6 nm, whereas residues 45–60 and 80–90 were closest to the surface. The two latter sequence parts correspond to the switch I and switch II regions, respectively. Rab(GTP) was more distant from the membrane surface right from the beginning with the largest distance of 6.0 nm around residue 80 (Figure 4(B)). During the 450 ns of simulation the overall protein–membrane distance decreased. The switch from a perpendicular position to the tilted orientation was observed after ∼200 ns. The distance–residue behaviour showed an inverse profile compared to Rab(GDP), i.e. residues that were at largest distance from the surface in the GDP-bound protein were closer in the GTP-bound protein and *vice versa*. Consequently, residues 50–60 and 75–92, which roughly represent the switch I and switch II regions, are more distant to the membrane surface in Rab5(GTP). As expected, residues from the C-terminus of Rab5 are very close to the bilayer or even insert into the bilayer, as shown by negative distances. For both, Rab5(GDP) and Rab5(GTP), residues at the extreme N-terminus are close to the bilayer as well and contribute to the Rab5-membrane stabilisation.

**Figure 5.**
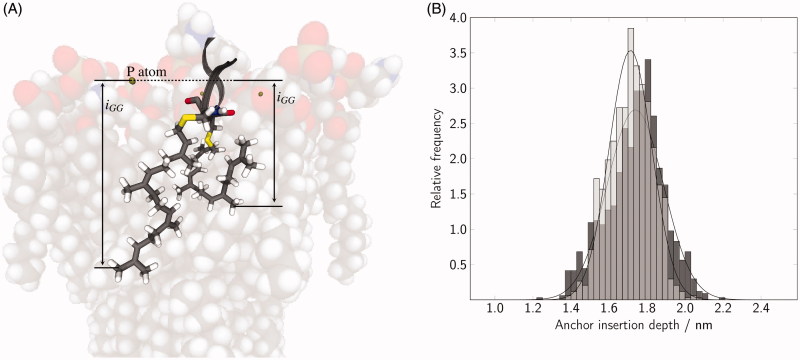
(A) The insertion depth of the GG chains within the bilayer was calculated as the z-distance between the neighbouring lipid P atoms and the most deeply inserted carbon atom of the GG chains. (B) The distribution of the anchor insertion depth in three averaged runs is shown for Rab(GDP) (black) and Rab(GTP) (gray).

For Rab(GDP), a larger number of C-terminal residues can be found in close proximity to the membrane surface compared to Rab(GTP). Therefore, Rab5(GDP) interactions with the membrane may be more pronounced than in Rab5(GTP). In order to probe whether this has any influence on the direct membrane anchoring via the two GG chains at the C-terminus, we investigated the insertion depth of the GG anchor chains. The anchor penetration was calculated as the z-distance between the lowest GG carbon atom and the phosphorous atoms of the surrounding phospholipid headgroups (Figure 5(A)). The insertion depths are normally distributed with average values of 1.74 nm for Rab(GDP) and 1.71 nm for Rab(GTP). These values correspond to approximately 38% of the overall bilayer thickness. The insertion depths were expected to vary only marginally with the nucleotide since NMR studies of the small GTPase Ras showed that the lipid anchors adapt to the bilayer thickness in order to avoid unfavourable hydrophobic mismatches^44^. Therefore, the insertion depth rather depends on the membrane structure than on the activation state of Rab5.

**Figure 6.**
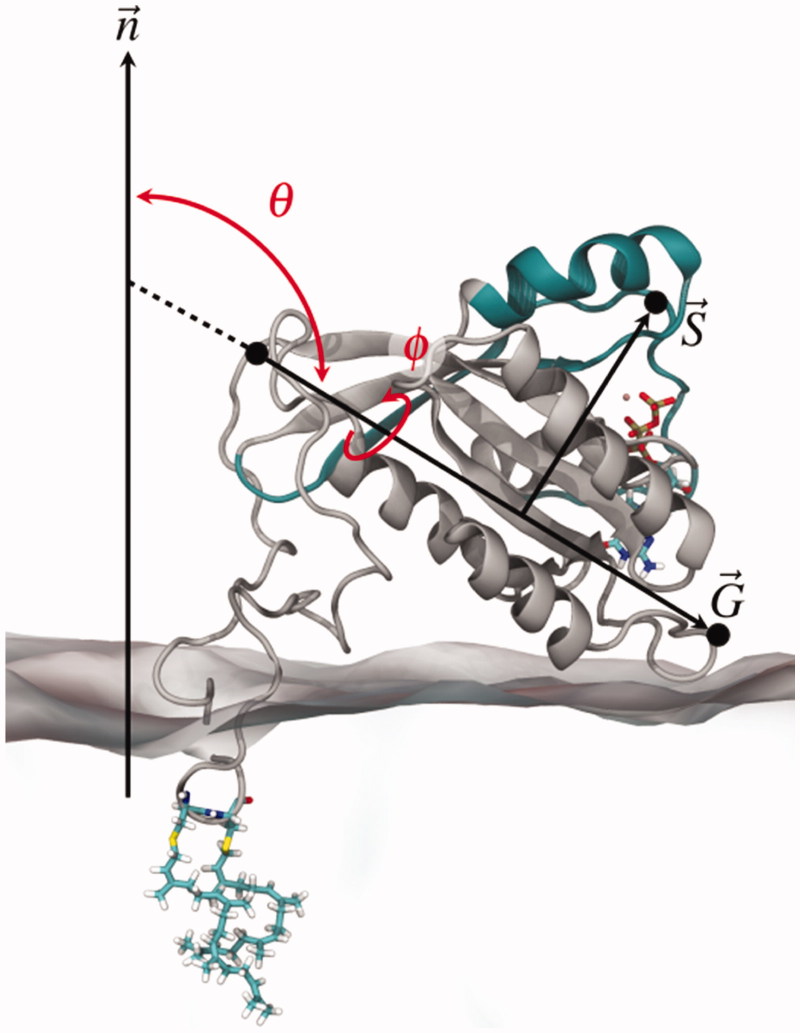
Two observables describe the Rab5 orientation at the membrane: the pivot angle *θ* reflecting the G domain orientation, → G, and the dihedral *φ* depicting the torsion between membrane, G domain and the switch regions.

Since the switch regions distance to the membrane surface was observed to differ significantly between Rab(GDP) and Rab(GTP), together with the overall protein–membrane distance, we investigated the relative protein orientations. The protein orientation with respect to the bilayer surface was defined by the pivot angle *θ* between the membrane normal, → n, and the direction vector of the protein G domain, → G (Figure 6). In addition, the dihedral *φ* describes the torsion angle between → n, → G, and the vector depicting the position of the switch regions relative to the G domain, → S. The dihedral *φ* allows distinguishing between a “syn” and an “anti” conformation of the switch regions, either facing the membrane surface or are solvent-exposed, respectively.

**Figure 7.**
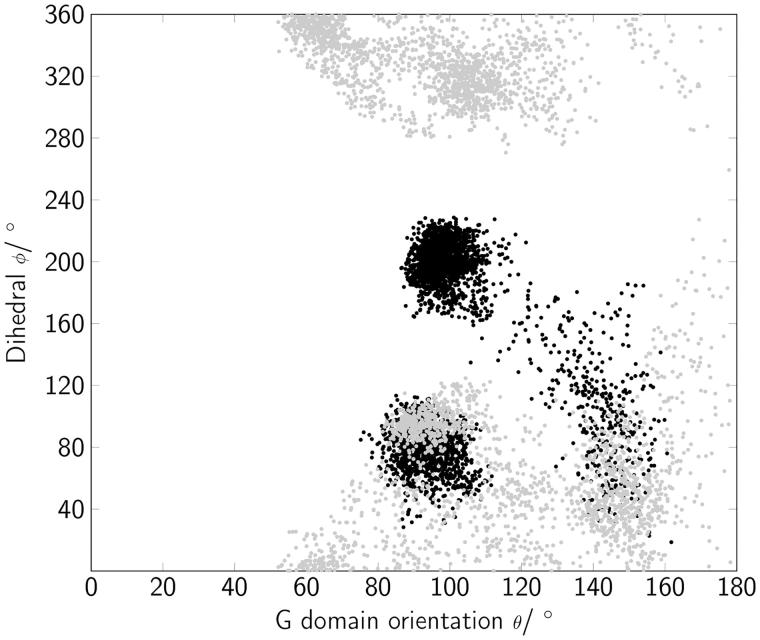
The orientation of Rab5 is described by the internal protein torsion as a function of the G domain orientation with respect to the membrane surface. Black dots correspond to positions of Rab(GDP); Rab(GTP) orientations are shown in gray.

The angle *θ* that was defined between the G domain main axis and the membrane normal varied in the range of 50° ≤ *θ* ≤ 180° (Figure 7). The spectrum was broader for Rab(GTP) than for Rab(GDP), while in the latter state *θ* varied between 80° and 110°. A pivot angle *θ* of 90° corresponded to a perfect parallel G domain orientation with respect to the membrane surface. The internal torsion of the protein regions *φ* showed two distinct populations for Rab(GDP): one was at ∼80° and the other population was at around 190°. A dihedral *φ* of 180° represents a “syn” conformation of the switch regions, i.e. the switch regions pointing towards the membrane surface. In contrast, for Rab(GTP) the dihedral *φ* adopted values between 0 and 90° as well as 280° ≤ *φ* ≤ 360°. Therefore, Rab5(GTP) was mainly found with its switch regions solvent-exposed, pointing towards the cytoplasm opposite to the membrane bilayer or laterally along the G domain.

**Figure 8.**
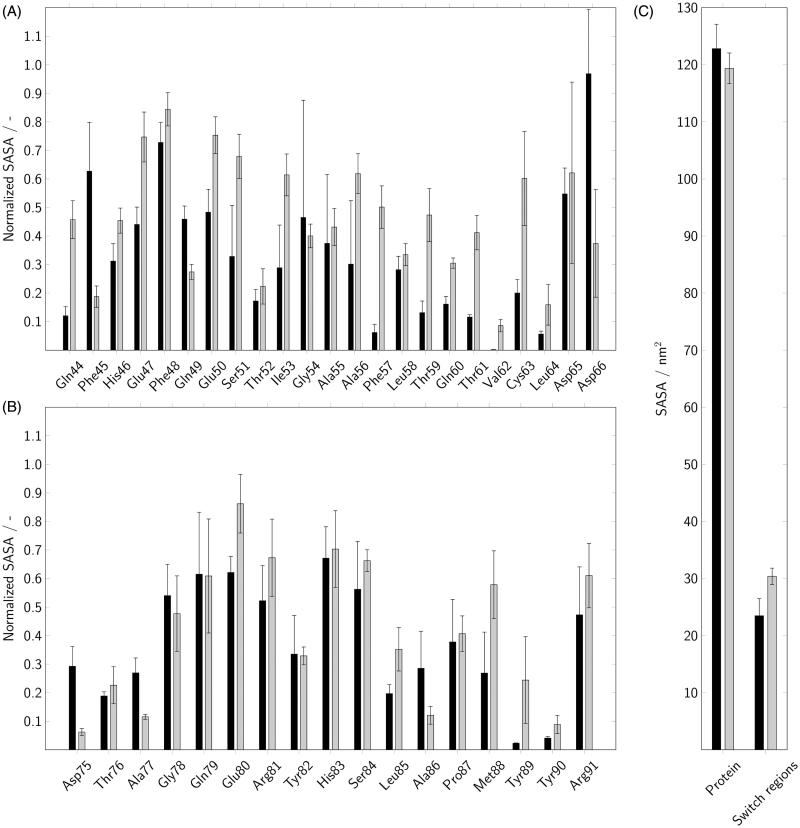
The normalised solvent-accessible surface area (SASA) of Rab5 was calculated for individual amino acids in the switch I (A) and switch II (B) regions. In addition, the overall SASA was determined for the whole protein as well as for both switch regions (C). Data for Rab(GDP) and Rab(GTP) are coloured in black and gray, respectively.

In order to verify if the switch regions in Rab5(GDP) are actually less solvent-exposed than in Rab(GTP), we calculated the solvent-accessible surface area (SASA) using VMD^45^ and a solvent probe radius of 1.4 Å (Figure 8). The SASA of the individual amino acids in the switch regions was normalised to the total accessible surface area for the corresponding residue “X” determined experimentally by Miller et al. in a Gly-X-Gly tripeptide^46^. A normalised SASA of 1 suggests almost complete water accessibility, i.e. the amino acid is sterically unhindered by adjacent residues or lipid molecules. Moreover, the total SASA of the protein as well as of both switch regions gives an idea of the spatial motility of the protein or the switch regions, respectively, as a whole.

**Figure 9.**
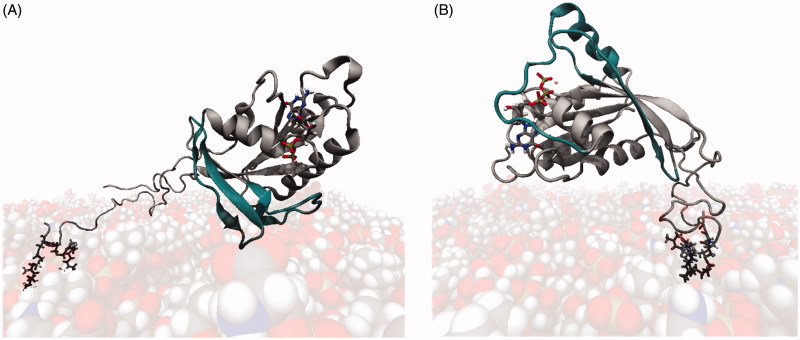
Exemplary final orientations of Rab5(GDP) (A) and Rab5(GTP) (B) after 500 ns of MD simulation. The proteins tilted towards the membrane surface with the switch regions (dark petrol) only partly accessible in Rab5(GDP) and solvent exposed in Rab5(GTP).

Except for Phe45 and Asp66, all residues in the switch I region showed a higher SASA in Rab5(GTP) compared to the inactive Rab5(GDP) (Figure 8(A)). The differences are most pronounced for Ser51, Ile53, Ala56, Phe57, Thr59, and Thr61 to Leu64 for which Rab(GDP) had less than 50% of the solvent exposed area of Rab(GTP). Error bars indicate the fluctuations between the three individual runs. Thus, the Rab(GDP) trajectories differed most for Gly54, which exhibited a large SASA in one specific simulation but not in the other two. In the switch II region, Rab5(GTP) also showed larger SASA values but with less significant differences (Figure 8(B)). The SASA of residues Met88 to Tyr90 was twice as high in Rab(GTP) compared to Rab(GDP). For both switch regions, the total area accessible to water molecules was 23.5 nm^2^ in Rab(GDP) and 30.4 nm^2^ in Rab(GTP) (Figure 8(C)). Surprisingly, the overall solvent accessibility of the protein was slightly higher in Rab(GDP) with 122.8 nm^2^ than in Rab(GTP) (119.4 nm^2^). This may be due to the more extended structure of the hypervariable linker region at the C-terminus of Rab5(GDP) and the larger distance from the membrane surface (Figure 9(A)).

## Discussion and conclusion

Three independent full-atomistic 450 ns MD simulations were performed for Rab5(GDP) and Rab5(GTP), respectively, yielding a total production simulation time of 1.35 µs for each nucleotide state. The three replicas showed similar qualitative protein behaviour with minor deviations concerning the orientation of individual amino acid residues. The long hypervariable C-terminal linker region allows a great degree of flexibility which is expected for a peripheral membrane protein. During the long simulation time, the G domain of both Rab(GDP) and Rab(GTP) re-orients from an initial perpendicular toward a tilted orientation. This is accompanied by a rotation of the switch regions around the G domain axis. For inactive Rab5(GDP), the switch regions are adopting a ‘syn’ configuration directly facing the membrane surface. Simultaneously, the G domain arranges almost parallel to the lipid bilayer so that the switch regions of Rab(GDP) are partially buried between protein and bilayer. In contrast, the switch regions of Rab(GTP) adopt an orientation in which they are fully solvent and effector accessible.

Our findings are in agreement with Gorfe et al., who performed all-atom MD simulations of full-length H-Ras^23^. They found two (possibly) nucleotide-dependent binding modes which differ regarding protein orientation and the contacts made between membrane and residues in the catalytic domain of Ras. Formation of stable contacts between the G domain and the bilayer could not always be observed within the 40 ns simulations of this study, which is why a definite assignment of the two orientations to either the GDP- or GTP-loaded state could not be made. For Rab5, a stable interaction between the G domain and the membrane phosphates was established after ∼120 ns in Rab(GDP) and after 200 ns in Rab(GTP) in our work. In a recent study, Prakash et al. investigated the orientational flexibility of membrane-bound full-length K-Ras in its GTP-bound form^24^. Our results agree with their finding of two dominant orientations characterised by the differing accessibility of the switch regions. One orientation with rather inaccessible switch regions featured a more extended structure of the hypervariable C-terminal region, which is in agreement with our Rab(GDP) structures. Distinct membrane orientations were previously described for the Ras superfamily of proteins. Different dominant membrane orientations for lipidated small GTPases (e.g. Ras, Rheb, and Arf1) and the general concept of membrane orientation dynamics were recently reviewed^25^. The small GTPase accessibility to downstream binding partners and protein signalling are discussed. This indicates that the G-domain of many if not all small GTPases generally forms different membrane interactions depending on their nucleotide state. We are here able to show that only the Rab(GTP) state displays solvent, effector protein and small molecule accessible switch regions which reveals the druggability of Rab or its effector proteins^47^.

The switch regions of small GTPases are markers of the nucleotide-bound state of the enzyme and essential for binding effectors and regulatory proteins. In the large human Rab protein family, the highest conservation of electrostatic potential is observed for residues of the two switch I and II regions^5^. The hydrophobic interaction, however, is short range, more specific and variable among the Rab proteins. This points to a long-range electrostatic recognition of the nucleotide-state and directed recruitment of Rab effector proteins and a specific short-range Rab-type adaptation.

One example is active Rab(GTP) that binds the C2H2 zinc finger of early endosome antigen 1 (EEA1) in order to mediate fusion of endosomes. Rab5 residues described to form contacts between both proteins are located in the switch regions; specifically Ile53, Ala55, Ala56, Phe57, Trp74, Tyr82, Leu85, Met88, and Arg91^48^. However, Rab5–protein interactions are not limited to Rab5(GTP) but do also occur in the Rab5(GDP) state. The guanosine nucleotide exchange factor (GEF) Rabex-5 induces the exchange of GDP with GTP in order to activate the Rab5. A fragment of nucleotide-free Rab5 has been crystallised earlier together with Rabex-5 and the effector Rabaptin-5^49^. Residues that are essential for the interaction between Rab5 and Rabex-5 are mainly located close to or within the Rab5 switch II region, namely Glu72, Asp75, Arg81, and Tyr89. Another example for Rab5(GDP) binding is the interaction with the Rab GDP dissociation inhibitor (GDI). GDI mediates the release of Rab(GDP) from the membrane and its stabilisation in the cytoplasm. Isothermal titration calorimetry measurements with yeast Ypt1 and GDI revealed three interaction sites in GDI^50^.

The Rab binding platform (RBP) forms contacts with multiple residues in the Rab5 switch I and II regions. Comparison of the structures of human Rab5(GDP) and Ypt1 reveals conserved interaction residues (see Figure S2, Supplementary Information). Ypt1 residues found to interact with GDI have their corresponding counterparts in Rab5, namely Ile53, Gly54, Ala56, Phe57, Trp74, Asp75, Ala77, Gln79, Tyr82, Ser84, Ala86, Pro87, Met88, and Arg91. The long flexible C-terminus of Rab5 binds to the GDI surface and third, the geranylgeranyl anchors interact with a hydrophobic GDI prenyl binding pocket. The need for accessible switch regions even in Rab5(GDP) is not in conflict with the protein orientations observed in MD simulations. The protein positioning and orientation is variable due to the very flexible linker region.

We speculate that approaching binding partners may induce a small re-orientation of Rab5 such that interactions with the switch region residues become possible. Our forthcoming MD simulations with human membrane-bound Rab5(GDP) associated with GDI intend to prove that a spontaneous re-orientation of the tilted protein switch regions may be possible. Such a re-orientation is necessary for the GDI hydrophobic pattern to incorporate the GG anchor chains^50^.

The discussed tilted Rab5 G domain orientation and protein–membrane interaction is necessary for all downstream processes, e.g. membrane tethering and fusion, Rab5 release from the membrane, or modifications of the lipid composition by phosphatidylinositol 3-kinases or phosphatases, to occur at or in close proximity to the bilayer. The specific interaction of membrane-bound Rab(GTP) with particular types of phospholipids will be discussed in a separate publication. It requires an exhaustive sampling and long timescale simulation of phospholipid diffusion enrichment. The spatial and temporal modification of membrane compositions in endocytosis is a trigger for the transformation from early to late endosome. The increase of negatively charged lipids like PI(3)P in late endosomes indicates the relevance of strong electrostatic interactions between membrane-bound Rab5 and Rab7 proteins and membrane lipids.

Additional MD simulations provide the opportunity to analyse the downstream interactions of Rab5 with its effector and regulatory proteins at the molecular level. Further we have given only two representative examples.

*Legionella pneumophila*, causing Legionnaires’ disease was shown to localise to early endosomes. Recent crystal structures revealed that it senses the GTP-bound state of Rab5 and cannot bind to Rab5 in its GDP-bound state. In order to localise and associate with early endosome membranes, VipD needs to outperform physiological effectors for Rab5 binding. EEA1, Rabaptin-5 and Rabenosyn-5 bind to Rab5 via a surface that includes the switch and interswitch region and that significantly overlaps with the epitope for VipD binding^51^.

Recent work has identified the Rab5 isoprenoid biosynthesis to be a valid target against malaria transmitted by *Plasmodium falciparum*. Inhibition of the prenylation pathway with the drug fosmidomycin leads to a loss of Rab5 localisation and Rab-mediated vesicular trafficking^52^.

Thus, as a key regulator of a broad spectrum of processes related to endocytosis, cellular trafficking and recycling vs. degradation events, Rab5 is of utmost importance within every single cell. This makes Rab5 very interesting as a drug target. Blocking specific effector interactions would most of the time result in the blocking of a large number of downstream interactions since signalling events are often build on complex cascades with positive feedback loops.

Since Rab proteins are members of the Ras GTPase superfamily, similar therapeutic approaches for diseases associated with Rab proteins may be taken. These comprise the direct inhibition of Ras, the interference with Ras-membrane association mechanisms, the inhibition of Ras downstream effectors, the synthesis of lethal components interacting specifically with mutant Ras proteins, and the investigation and utilisation of Ras-induced changes in cell metabolism^16^. Pharmaceutical industry has developed approaches to inhibit the farnesyl transferase and, after the discovery that K-Ras was also geranylgeranylated, to identify geranylgeranyl transferase inhibitors^53^. GEFs and GAPs lack pronounced binding pockets at the active sites for small molecules which obstructs them to be druggable by small molecules. However, there is evidence that GEFs and perhaps GAPs can be targeted by interfacial “inhibition” or even “stabilisation” which prevents the activation (for a review, see^53^). Similar approaches can be expected to be feasible for Rab proteins.

## Supplementary Material

IENZ_1260564_Supplementary_Material.pdf
