# Supplementary material for: Probing the druggability of membrane-bound Rab5 by molecular dynamics simulations
Source: J Enzyme Inhib Med Chem. 2017 Jan 16;32(1):434–43. doi: 10.1080/14756366.2016.1260564 (PMC6010109; doi:10.1080/14756366.2016.1260564)
Supplement: IENZ_1260564_Supplementary_Material.pdf [file IENZ_A_1260564_SM4226.pdf]

Supplementary Information

"The Druggability of Membrane-Bound Rab5 Investigated by Molecular Dynamics Simulations"

Eileen Edler[1] and Matthias Stein[1*]

[1] Max Planck Institute for Dynamics of Complex Technical Systems, Molecular Simulations and Design Group, Sandtorstrasse 1, 39106 Magdeburg, Germany.

* Corresponding author: Dr Matthias Stein, email: Matthias.stein@mpi-magdeburg.mpg.de, fax +49 391 6110 436

Protein structures

Pdb structures of full-length Rab5(GDP) and Rab5(GTP) GG-anchored to a six-component membrane are provided

Six-component membrane equilibration

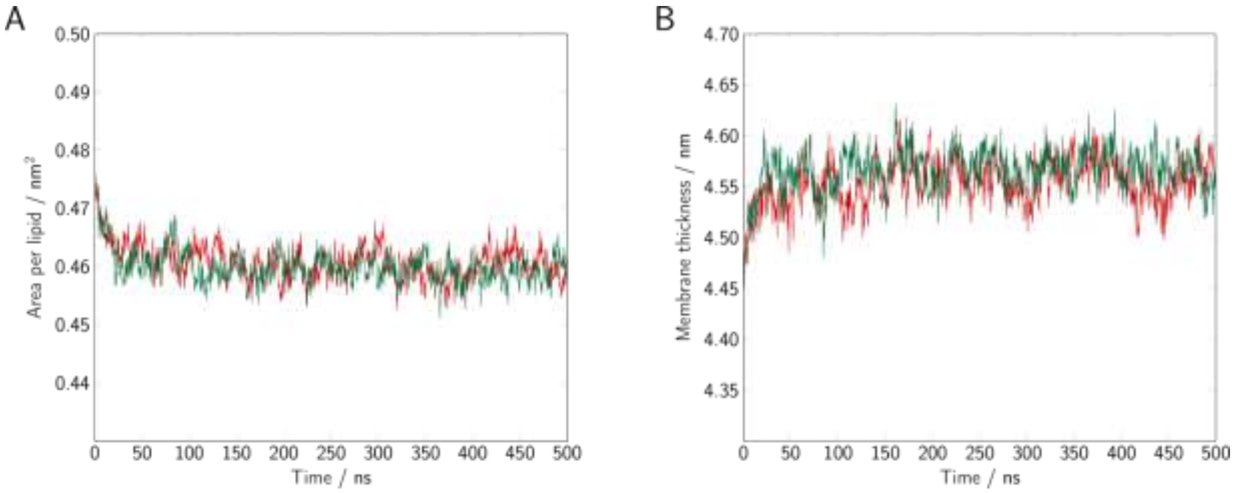

Figure S1: Area per lipid in nm^2 (**A**) and membrane thickness in nm (**B**) were monitored over the whole simulation time to ensure sufficient equilibration of the lipid bilayer. The parameters for one out of three representative trajectories of Rab5(GDP) and Rab5(GTP) are shown in red and green, respectively.

Comparison of GDI-binding residues of human Rab5(GDP) and yeast Ypt1

Rab5	Ypt1
Ile53	Ile41
Gly54	Gly42
Ala56	Asp44
Phe57	Phe45
Trp74	Trp62
Asp75	Asp63
Ala77	Ala65
Gln79	Gln67
Tyr82	Phe70
Ser84	Thr72
Ala86	Thr74
Pro87	Ser75
Met88	Ser76
Arg91	Arg79

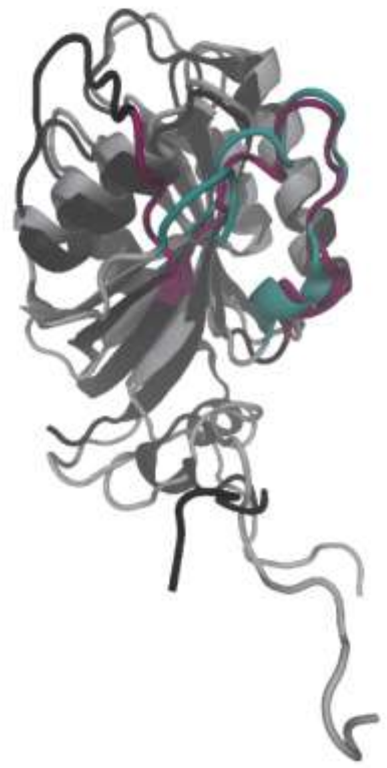

Figure S2: Residues the G-domain of human Rab5(GDP) and Ypt1 that form contact between the small GTPase and the Rab-binding GDI. Right: Structural superposition of cytoplasmic human Rab5 (grey) and Ypt1 (black) with the interaction-forming residues highlighted in dark petrol or purple, respectively.
